# Supplementary material for: Alternate subunit assembly diversifies the function of a bacterial toxin
Source: Nat Commun. 2019 Aug 15;10:3684. doi: 10.1038/s41467-019-11592-0 (PMC6695444; doi:10.1038/s41467-019-11592-0)
Supplement: Supplementary file 1 — Supplementary Information [file 41467_2019_11592_MOESM1_ESM.pdf]

Supplementary Information

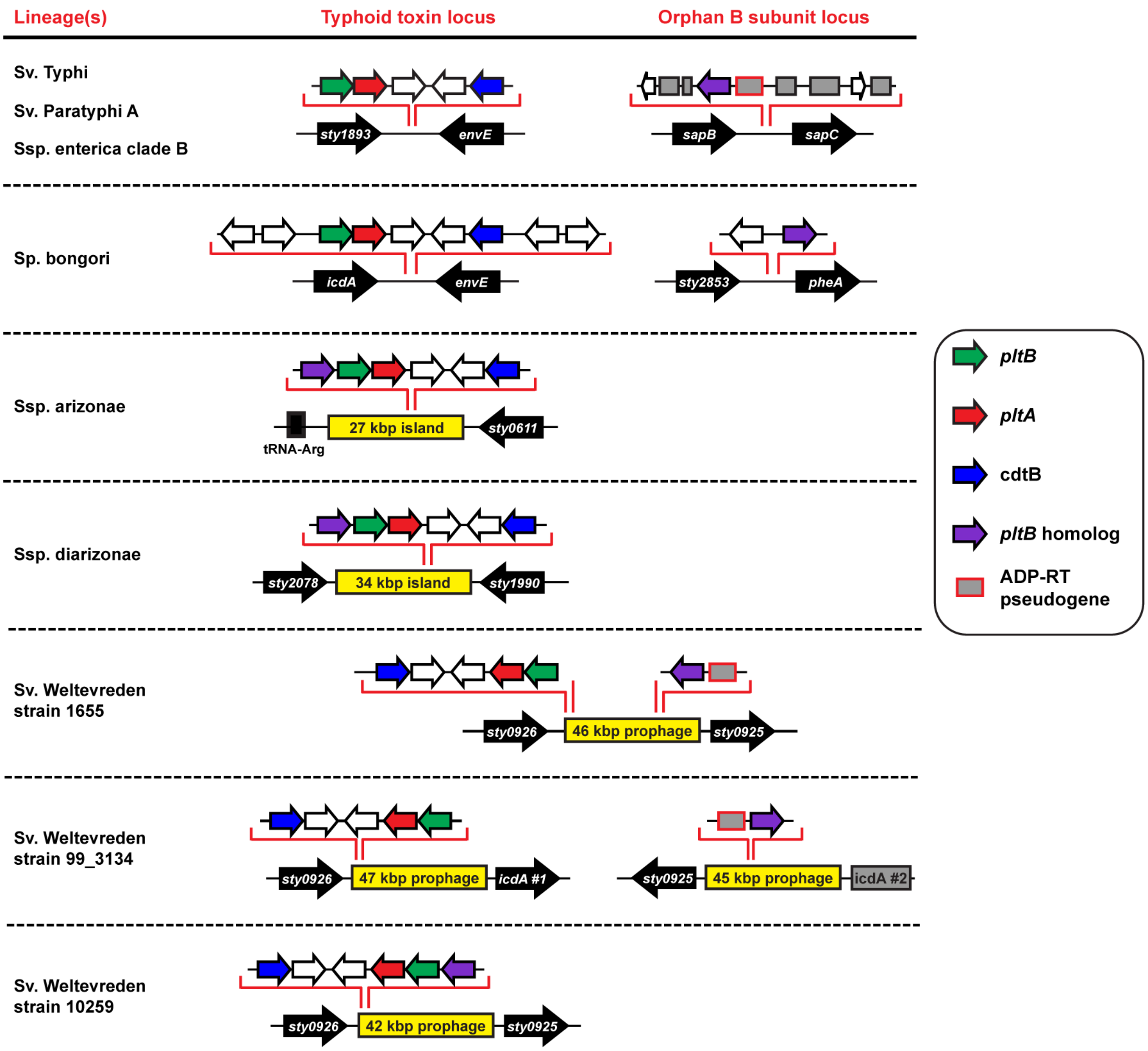

Supplementary Fig. 1. Congruent phylogenetic distributions of the typhoid toxin islet and a second pertussis toxin-like B subunit homolog in the *Salmonella* genus. Depiction of different genomic arrangements of the typhoid toxin islet and of loci encoding a second putative B subunit that were identified by searching the NCBI genome database. All genomic locations are shown relative to the *S*. Typhi reference genome (black arrows). Large genomic islands absent from the Typhi genome are shown in yellow, pseudogenes are shown as grey boxes and ADP-ribosyltransferase (ADP-RT) pseudogenes have a red outline. The depicted genomic arrangements appear, based on the available sequence data, to be conserved across the cited species (Sp.), subspecies (Ssp.), clade, serovar (Sv.) or strains, as indicated.

```
PltB: MYMSKYVPVYTLLILIYSFNASAEWTGDNTNAYYSDEVIS
PltC: --MKKKLKVLTLALASISSVCYAAMADYDT--YVSNVQIN

PltB: ELHVGQIDT----SPYFCIKTVKANGSGTPVVACAVSKQS
PltC: NLSYGVYTSGGKETQFFCIGLKHGSEAISINAMCKVDVYG

PltB: IWAPSFKELLDQARYFYSTGQSVRIHVQKNIWTYPLFVNT
PltC: NHKQGFDNMLNTAKYYYTTGGDVRIYYKENVWRDPDFKSA

PltB: FSANALVGLSSCSATQ-CFGPK
PltC: FSSRELIAITTCSSSSYCMGPTVTN
```

40/145 identical (28%) // 68/145 similar (47%)

Supplementary Fig. 2. Sequence alignment of *S*. Typhi PltB and PltC. Amino acid sequence comparison between *S*. Typhi PltB and PltC (Sty1364). A pairwise global alignment was done using the EMBL-EBI Matcher program (https://www.ebi.ac.uk/Tools/psa/emboss_matcher/nucleotide.html)

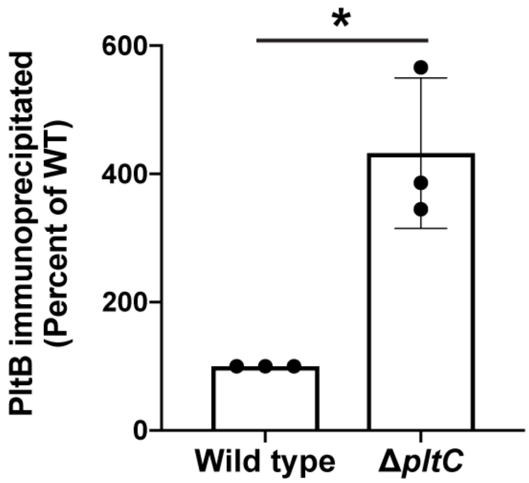

Supplementary Fig. 3. Deletion of *pltC* results in increased production of PltB-typhoid toxin. Two CdtB-3xFLAG encoding *S*. Typhi strains, wild type and Δ*pltC*, were grown in TTIM to induce typhoid toxin expression. Cell lysates were immunoprecipitated with an anti-FLAG antibody and the abundance of PltB and CdtB in the eluates was measured by western blot using anti-PltB and anti-FLAG antibodies, respectively. The abundance of PltB in the wild-type and Δ*pltC* samples was normalized to the CdtB levels and compared using a one-sample t-test to determine if PltB abundance in the Δ*pltC* sample deviated from a theoretical value of 100% of the wild-type value. Mean values +/- S.D. are shown for 3 independent experiments. * $p < 0.05$.

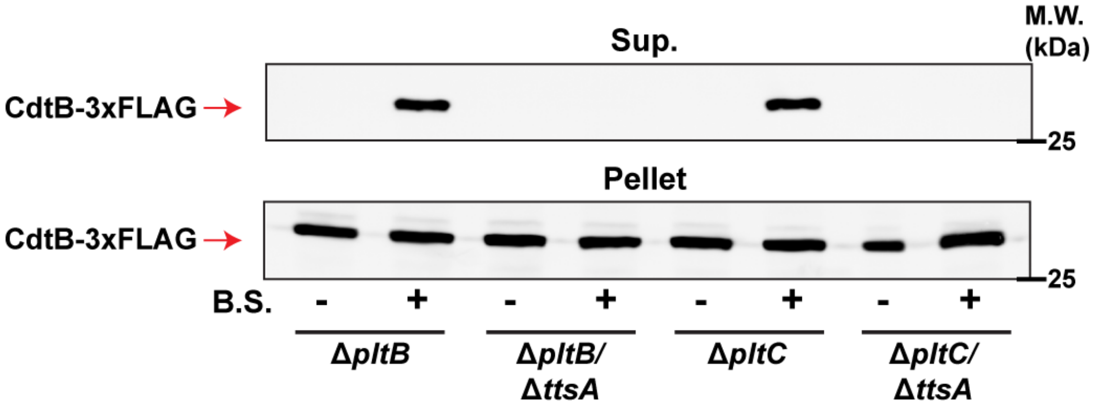

Supplementary Fig. 4. Both typhoid toxins are secreted using the same TtsA-dependent secretion mechanism. The mechanism of typhoid toxin secretion was recently described, in which the activity of the specialized muramidase TtsA enables the toxin to access the trans side of the peptidoglycan layer, after which it can be released following exposure to outer membrane perturbing agents such as bile salts [1]. To determine whether this mechanism is utilized by both PltC-typhoid toxin and PltB-typhoid toxin, an *in vitro* secretion assay was performed [1]. The indicated strains, all of which encode a 3xFLAG-epitope tagged CdtB, were grown for 24 hours in TTIM medium to induce typhoid toxin (and *ttsA*) expression, after which the bacteria were pelleted and washed thoroughly. The material was evenly divided between two samples, one of which was incubated in PBS containing 0.075% bile salts (B.S.) and the other in PBS only. Samples were then pelleted and the levels of CdtB-3xFLAG in the pellet fraction and in filtered culture supernatants (Sup.) were analyzed by western blot. Source data are provided as a Source Data file.

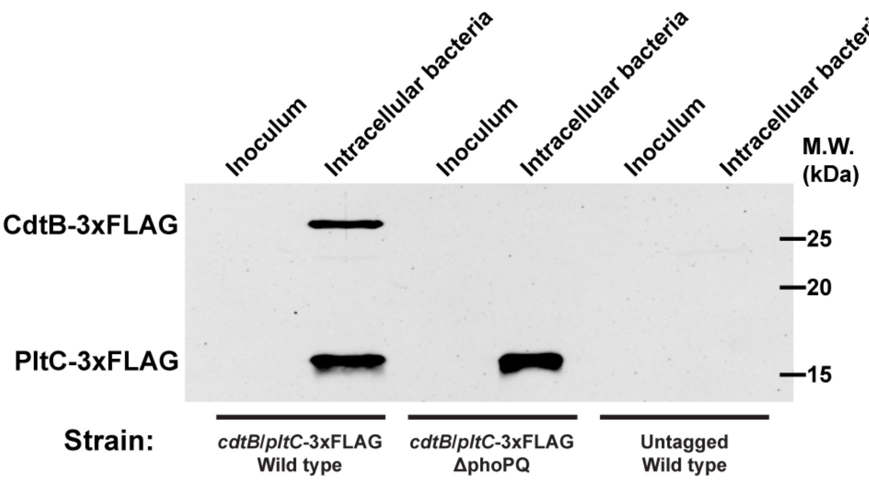

Supplementary Fig. 5. Expression of *pltC* by intracellular *S*. Typhi, unlike the other typhoid toxin genes, does not require the PhoP/PhoQ two-component system. Henle-407 cells were infected using wild type and Δ*phoP/phoQ* mutant *S*. Typhi strains that encode 3xFLAG epitope-tagged versions of both *cdtB* and *pltC* at their native genomic loci, as well as an untagged control strain. Whole cell lysates from the inoculum used for the infections as well as the bacteria isolated from infected cells 24 hours post-infection were analyzed by western blot. Sample loading was normalized to CFU recovery and lysate from $2.5x10^7$ bacteria was loaded in each lane. Source data are provided as a Source Data file.

a

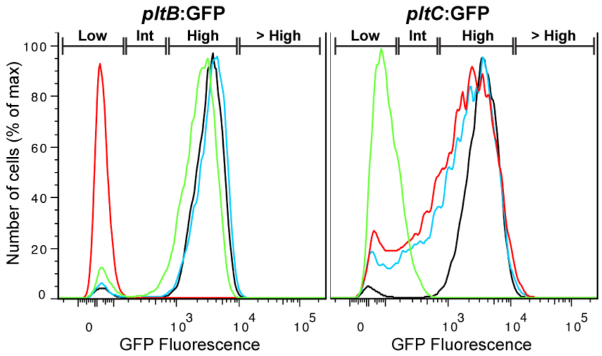

	Low	Int	High	> High	Low	Int	High	> High
**WT**	2.4	0.6	96.9	0.1	2.3	1.2	96.3	0.1
ΔphoP/phoQ	98.6	1.4	-	-	8.8	22.5	67.4	0.6
ΔssrA/ssrB	5.3	4.1	90.6	-	71.0	25.0	0.1	-
ΔenvZ/ompR	3.0	1.9	95.9	0.2	7.1	19.7	72.3	0.4

b

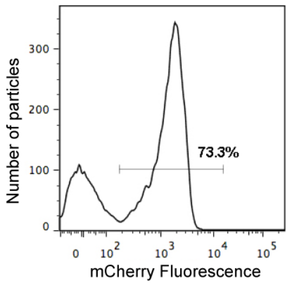

Supplementary Fig. 6. Single bacterium-level expression of the typhoid toxin B subunits by *S*. Typhi within infected cells in strains lacking key two-component regulators of intracellular gene expression. (**a**) The SsrA/SsrB, PhoP/PhoQ and EnvZ/OmpR two-component regulatory systems are all central regulators of intracellular gene expression and all were identified as leading to reduced *pltC* expression in the FAST-INseq screen (Supplementary Table 3). To probe the role of these regulators in the expression of *pltB* and *pltC*, a flow cytometry analysis of *pltB:gfp* and *pltC:gfp* expression was conducted of the indicated *S.* Typhi strains 24 hours post-infection. Histograms show the GFP fluorescence intensities of individual bacteria. For both wild-type strains, samples show high fluorescence (most bacteria) and low fluorescence (rare bacteria) populations; gates were established to show the percentage of bacteria in these populations for each strain, as well as those with an intermediate level of fluorescence ("int") and those with a level greater than 99.9% of the wild-type population (> high). The proportions of bacteria with fluorescence intensities within these gates is shown (bottom). The heterogeneous expression of *pltC* in the Δ*phoP*/*phoQ* and Δ*envZ*/*ompR* strains is likely due to their influence over *ssrA*/*ssrB* expression[2,3]. (**b**) Gating strategy used for flow cytometry to analyze *S*. Typhi typhoid toxin gene expression at the single bacterium level during infection (panel (**a**), Fig. 3e). Strains carried a low copy plasmid that constitutively expresses mCherry. To identify particles that represent *S*. Typhi (as opposed to cell debris remaining from host cell lysis), only particles with an mCherry fluorescence level above background were analyzed. An example of this gating (*pltC*:GFP wild-type strain) is shown.

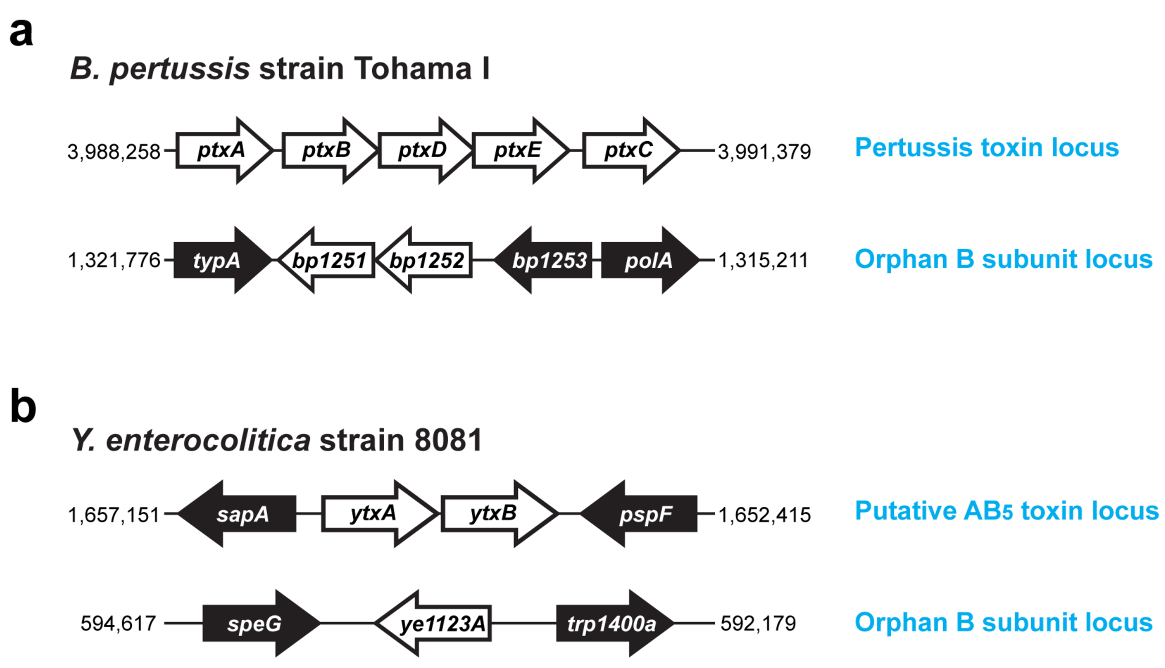

Supplementary Fig. 7. Examples of AB_5-type toxins and orphan homologous delivery subunits encoded within the same genome outside the *Salmonella* genus. Using BLAST homology searches using AB_5-type toxin subunits, genomes encoding an AB_5-type toxin as well as a homologous orphan delivery subunit were identified and are depicted above. (**a**) In addition to the five-gene pertussis toxin locus [4], we found that *B. pertussis* encodes two putative AB_5 toxin delivery subunits at a distant genomic locus that does not also encode a putative toxin active subunit, *bp1251* and *bp1252*. Bp1251 is >30% identical to both PtxB and PtxC over >200 amino acids. Bp1252 was identified as a statistically significant hit (e value < 0.02) to PtxB, PtxC and PtxE using the HHPred protein homology detection tool (https://toolkit.tuebingen.mpg.de/#/tools/hhpred). (**b**) The putative AB_5-type toxin Yersinia toxin [5] is encoded by *ytxA* and *ytxB* in *Y. enterocolitica*. At a distant genomic location, *Y. enterocolitica* also encodes an orphan pertussis toxin-like delivery subunit, *ye1123A*. YtxA and Ye1123A exhibit significant sequence similarity to the pertussis-like toxin ArtAB; YtxA is > 60% identical to ArtA and Ye1123A is ~30% identical to ArtB.

Supplementary Table 1: Genes identified by FAST-INSeq as important for *pltC* expression in infected human cells

Gene	High fluor: insertions	High fluor: reads	Low fluor: insertions	Low fluor: reads	Ratio[a]
			Gene regulation		
ssrB	22	48	27	936	20
ssrA	36	52	61	839	16
phoP	13	33	15	201	6
phoQ	26	62	27	335	5.5
ompR	14	73	13	244	3.5
			Pyrimidine biosynthesis		
carA	10	98	11	897	9
carB	35	194	33	1669	8.5
pyrD	21	139	20	979	7
pyrE	19	477	16	2705	5.5
pyrB	19	122	16	536	4.5
pyrF	10	40	7	188	4.5
			Other		
proB	20	307	20	2449	8
proC	12	216	11	1343	6
rplS	3	45	3	238	5.5
proA	27	798	24	3704	4.5
ugpQ	15	677	13	2652	4
t3900 (bcsZ)	25	260	19	864	3.5
lysA	33	1004	30	3497	3.5
t4432 (ulaR)	16	86	8	308	3.5
vexA	33	1099	25	3216	3
t3052	13	549	10	1292	2.5
tviB	45	1990	35	5021	2.5

[a]: Ratio of normalized INSeq sequencing reads from transposon insertions within the indicated gene in the low fluorescence pool compared to the high fluorescence pool.

Supplementary Table 2: Mutants identified by FAST-INSeq that lead to an increased fraction of *S*. Typhi expressing high levels of *pltC* in infected human cells

Gene	High fluor: insertions	High fluor: reads	Low fluor: insertions	Low fluor: reads	Ratio[a]
			Biotin biosynthesis		
bioC	12	289	7	46	6.5
bioA	31	679	20	131	5
bioD	16	436	9	90	5
bioF	27	929	19	195	5
bioB	33	639	22	150	4
bioH	19	1180	13	329	4
			Purine metabolism		
purD	13	328	4	25	13
apt	12	158	7	21	7.5
purM	17	321	7	60	5.5
cpdA	24	1432	16	455	3
			Other		
t3263	9	198	4	15	13
ssaQ	23	142	6	16	9
ilvY	9	200	3	22	9
tatB	9	154	2	17	9
rpmE2	12	167	7	19	9
t3867	6	204	3	35	6
t1909	18	279	9	46	6
t2225	6	435	4	77	5.5
t4475	6	244	4	46	5.5
t0644	16	285	10	54	5.5
sufI	29	615	12	118	5
hycF	8	221	5	45	5
t1519	25	263	14	61	4.5
t2471	18	561	12	151	4
t2824	7	403	6	112	4
dacA	18	411	9	106	4
kdpB	26	484	13	127	4
t3076	12	448	8	137	3.5
t4459	10	388	6	130	3

[a]: Ratio of normalized INSeq sequencing reads from transposon insertions within the indicated gene in the high fluorescence pool compared to the low fluorescence pool.

Supplementary Table 3: List of bacterial strains used in this study

Strain	Relevant genotype	Reference
ISP2825	Wild type *S*. Typhi	[6]
SB1946	*cdtB*-3xFLAG	[7]
SB2612	*pltB*:*lacZ*	[8]
SB2625	*pltB*:*lacZ* Δ*phoPQ*	[8]
SB2718	*pltB*:*gfp*	[8]
SB2900	*pltB*:*gfp* Δ*phoPQ*	[8]
SB2613	*cdtB*:*lacZ*	[8]
SB3211	*cdtB*-3xFLAG, Δ*pltC*	This study
SB3222	*cdtB*-3xFLAG, Δ*pltA*	This study
SB3226	*cdtB*-3xFLAG, Δ*pltB*	This study
SB3385	*cdtB*-3xFLAG, Δ*pltB,* Δ*pltC*	This study
SB3207	*pltC*-3xFLAG	This study
SB3406	*pltC*-3xFLAG, Δ*cdtB*	This study
SB3221	*pltC*-3xFLAG, Δ*pltA*	This study
SB3227	*pltC*-3xFLAG, Δ*pltB*	This study
SB3230	*malE*-3xFLAG (at *pltC* locus)	This study
SB3219	*pltC*-3xFLAG, *cdtB*-3xFLAG	This study
SB3229	*pltC*-3xFLAG, *cdtB*-3xFLAG, Δ*phoPQ*	This study
SB3679	cdtB-3xFLAG, Δ*ttsA*, Δ*pltC*	This study
SB3678	cdtB-3xFLAG, Δ*ttsA*, Δ*pltB*	This study
SB3210	Δ*pltC*	This study
SB3383	Δ*pltB*	This study
SB3384	Δ*pltC,* Δ*pltB*	This study
SB3407	*pltB*:*lacZ*, Δ*ssrAB*	This study
SB3654	*pltB*:*lacZ*, Δ*purM*	This study
SB3668	*pltB*:*lacZ*, Δ*pyrC*	This study
SB3220	*pltC*:*lacZ*	This study
SB3224	*pltC*:*lacZ*, Δ*phoPQ*	This study
SB3409	*pltC*:*lacZ*, Δ*ssrAB*	This study
SB3655	*pltC*:*lacZ*, Δ*purM*	This study
SB3669	*pltC*:*lacZ*, Δ*pyrC*	This study
SB3687	*pltB*:*gfp,* Δ*ssrAB*	This study
SB3675	*pltB*:*gfp,* Δ*envZ/ompR*	This study
SB3656	*pltB*:*gfp,* Δ*purM*	This study
SB3670	*pltB*:*gfp,* Δ*pyrC*	This study
SB3400	*pltC*:*gfp*	This study
SB3401	*pltC*:*gfp*, Δ*phoPQ*	This study
SB3688	*pltC*:*gfp*, Δ*ssrAB*	This study
SB3676	*pltC*:*gfp,* Δ*envZ/ompR*	This study
SB3657	*pltC*:*gfp*, Δ*purM*	This study
SB3671	*pltC*:*gfp*, Δ*pyrC*	This study

Supplementary References

1 Geiger, T., Pazos, M., Lara-Tejero, M., Vollmer, W. & Galán, J. Peptidoglycan editing by a specific LD-transpeptidase controls the muramidase-dependent secretion of typhoid toxin. *Nat Microbiol.* **3**, 1243-1254 (2018).

2 Fass, E. & Groisman, E. Control of Salmonella pathogenicity island-2 gene expression. *Curr Opin Microbiol.* **12**, 199-204 (2009).

3 Lee, A., Detweiler, C. & Falkow, S. OmpR regulates the two-component system SsrA-ssrB in Salmonella pathogenicity island 2. *J. Bacteriol.* **182**, 771-781 (2000).

4 Gross, R., Aricò, B. & Rappuoli, R. Genetics of pertussis toxin. *Mol Microbiol.* **3**, 119-124 (1989).

5 Axler-Diperte, G., Miller, V. & Darwin, A. YtxR, a conserved LysR-like regulator that induces expression of genes encoding a putative ADP-ribosyltransferase toxin homologue in Yersinia enterocolitica. *J Bacteriol.* **188**, 8033-8043 (2006).

6 Galan, J. E. & Curtiss, R., 3rd. Distribution of the invA, -B, -C, and -D genes of Salmonella typhimurium among other Salmonella serovars: invA mutants of Salmonella typhi are deficient for entry into mammalian cells. *Infect Immun* **59**, 2901-2908 (1991).

7 Spano, S., Ugalde, J. E. & Galan, J. E. Delivery of a Salmonella Typhi exotoxin from a host intracellular compartment. *Cell Host Microbe* **3**, 30-38, doi:10.1016/j.chom.2007.11.001 (2008).

8 Fowler, C. & Galán, J. Decoding a Salmonella Typhi Regulatory Network that Controls Typhoid Toxin Expression within Human Cells. *Cell Host Microbe* **23**, 65-76 (2018).
