# Supplementary material for: Alternate subunit assembly diversifies the function of a bacterial toxin
Source: Nat Commun. 2019 Aug 15;10:3684. doi: 10.1038/s41467-019-11592-0 (PMC6695444; doi:10.1038/s41467-019-11592-0)
Supplement: Supplementary file 3 — Description of Additional Supplementary Files [file 41467_2019_11592_MOESM3_ESM.pdf]

Description of Additional Supplementary Files

File Name: Supplementary Data 1
Description: Complete data set for immunoprecipitation-LC/MS/MS experiments

File Name: Supplementary Data 2
Description: Complete data set for FAST-INseq genetic screen
