# Supplementary material for: Alternate subunit assembly diversifies the function of a bacterial toxin
Source: Nat Commun. 2019 Aug 15;10:3684. doi: 10.1038/s41467-019-11592-0 (PMC6695444; doi:10.1038/s41467-019-11592-0)
Supplement: Supplementary file 6 — Reporting Summary [file 41467_2019_11592_MOESM6_ESM.pdf]

nature research

Corresponding author(s): Jorge E. Galan

Last updated by author(s): Jun 27, 2019

Reporting Summary

Nature Research wishes to improve the reproducibility of the work that we publish. This form provides structure for consistency and transparency in reporting. For further information on Nature Research policies, see Authors & Referees and the Editorial Policy Checklist.

Statistics

For all statistical analyses, confirm that the following items are present in the figure legend, table legend, main text, or Methods section.

n/a	Confirmed	
☐	☒	The exact sample size (*n*) for each experimental group/condition, given as a discrete number and unit of measurement
☐	☒	A statement on whether measurements were taken from distinct samples or whether the same sample was measured repeatedly
☐	☒	The statistical test(s) used AND whether they are one- or two-sided<br>*Only common tests should be described solely by name; describe more complex techniques in the Methods section.*
☐	☒	A description of all covariates tested
☐	☒	A description of any assumptions or corrections, such as tests of normality and adjustment for multiple comparisons
☐	☒	A full description of the statistical parameters including central tendency (e.g. means) or other basic estimates (e.g. regression coefficient) AND variation (e.g. standard deviation) or associated estimates of uncertainty (e.g. confidence intervals)
☐	☒	For null hypothesis testing, the test statistic (e.g. *F*, *t*, *r*) with confidence intervals, effect sizes, degrees of freedom and *P* value noted<br>*Give P values as exact values whenever suitable.*
☒	☐	For Bayesian analysis, information on the choice of priors and Markov chain Monte Carlo settings
☒	☐	For hierarchical and complex designs, identification of the appropriate level for tests and full reporting of outcomes
☒	☐	Estimates of effect sizes (e.g. Cohen's *d*, Pearson's *r*), indicating how they were calculated

Our web collection on statistics for biologists contains articles on many of the points above.

Software and code

Policy information about availability of computer code

Data collection	Micro-manager (https://micro-manager.org)
Data analysis	Graphpad Prism versions 7 and 8, Adobe Photoshop, Adobe Illustrator, Microsoft Excel, Micro-manager (https://micro-manager.org), FlowJo (FlowJo, LLC), INSeq_pipeline_v2 package (Andrew Goodman lab, Yale University).

For manuscripts utilizing custom algorithms or software that are central to the research but not yet described in published literature, software must be made available to editors/reviewers. We strongly encourage code deposition in a community repository (e.g. GitHub). See the Nature Research guidelines for submitting code & software for further information.

Data

Policy information about availability of data

All manuscripts must include a data availability statement. This statement should provide the following information, where applicable:

- Accession codes, unique identifiers, or web links for publicly available datasets
- A list of figures that have associated raw data
- A description of any restrictions on data availability

The data that support this study are included in this publication.

Field-specific reporting

Please select the one below that is the best fit for your research. If you are not sure, read the appropriate sections before making your selection.

☒ Life sciences ☐ Behavioural & social sciences ☐ Ecological, evolutionary & environmental sciences

For a reference copy of the document with all sections, see nature.com/documents/nr-reporting-summary-flat.pdf

Life sciences study design

All studies must disclose on these points even when the disclosure is negative.

Sample size	Sample sizes were empirically determined to optimize numbers based on our previous experience with equivalent experiments.
Data exclusions	No data were excluded
Replication	All findings described here were confirmed by repeating experiments and, when possible/applicable, performing distinct experiments to support the same experimental finding.
Randomization	Where applicable, samples and animals were randomly allocated into different groups.
Blinding	Investigators were not blinded to group allocation during the experiments or to the outcome assessment.

Reporting for specific materials, systems and methods

We require information from authors about some types of materials, experimental systems and methods used in many studies. Here, indicate whether each material, system or method listed is relevant to your study. If you are not sure if a list item applies to your research, read the appropriate section before selecting a response.

Materials & experimental systems

n/a	Involved in the study	
☐	☒	Antibodies
☐	☒	Eukaryotic cell lines
☒	☐	Palaeontology
☐	☒	Animals and other organisms
☒	☐	Human research participants
☒	☐	Clinical data

Methods

n/a	Involved in the study	
☒	☐	ChIP-seq
☐	☒	Flow cytometry
☒	☐	MRI-based neuroimaging

Antibodies

Antibodies used	M2 anti-FLAG mouse monoclonal (Sigma). Anti-CdtB rabbit polyclonal and anti-PltB rabbity polyclonal (this study).
Validation	Antibodies were all validated using multiple experiments wherein purified components and bacterial-derived samples were probed by western blot, seeking bands of the anticipated size for the target protein in positive controls, but lacking that same band in parallel samples that lack that target protein (such as lysates from isogenic deletion strains or purified toxins that lacking that component).

Eukaryotic cell lines

Policy information about cell lines

Cell line source(s)	Henle-407 human epithelial cell line, obtained from the Roy Curtiss laboratory collection
Authentication	The cells were frequently checked for their morphological features, growth propoerties and functionalities
Mycoplasma contamination	Cells were routinely tested for Mycoplasma contamination and tested negative.
Commonly misidentified lines (See ICLAC register)	No commonly misidentified cell lines were used

Animals and other organisms

Policy information about studies involving animals; ARRIVE guidelines recommended for reporting animal research

Laboratory animals	A mix of male and female 6-10 week old C57BL/6 mice were used in this study.
Wild animals	No wild animals were used in this study

Field-collected samples	No field-collected samples were used in this study
Ethics oversight	All animal experiments followed the ethical regulations and were conducted according to protocols approved by Yale University's Institutional Animal Care and Use Committee

Note that full information on the approval of the study protocol must also be provided in the manuscript.

Flow Cytometry

Plots

Confirm that:

☒ The axis labels state the marker and fluorochrome used (e.g. CD4-FITC).

☒ The axis scales are clearly visible. Include numbers along axes only for bottom left plot of group (a 'group' is an analysis of identical markers).

☒ All plots are contour plots with outliers or pseudocolor plots.

☒ A numerical value for number of cells or percentage (with statistics) is provided.

Methodology

Sample preparation	A detailed description is provided in the Methods section under the heading "Analytical Flow Cytometry"
Instrument	BD FACS Aria II
Software	FlowJo
Cell population abundance	Bacteria isolated from infected cells were analyzed. Bacteria constitutively expressed mCherry and were identified on the basis of their fluorescence in this channel.
Gating strategy	Bacteria constitutively expressed mCherry and were identified on the basis of their fluorescence in this channel. Gating was accomplished by setting a threshold in this channel above the level observed for any particles in parallel samples that lacked a source of mCherry.

☒ Tick this box to confirm that a figure exemplifying the gating strategy is provided in the Supplementary Information.
